# Application of RNA-Based Next-Generation Sequencing Fusion Assay for Hematological Malignancies

**DOI:** 10.3390/ijms26020435

**Published:** 2025-01-07

**Authors:** Fei Fei, Milhan Telatar, Vanina Tomasian, Lisa Chang, Mariel Gust, Hooi Yew, Tamerisa Dyer, Olga Danilova, Javier Arias-Stella, Raju Pillai, Ibrahim Aldoss, F. Marc Stewart, Pamela S. Becker, Vinod Pullarkat, Guido Marcucci, Michelle Afkhami

**Affiliations:** 1 Department of Pathology, City of Hope Comprehensive Cancer Center, Duarte, CA 91010, USA; ffei@coh.org (F.F.); mtelatar@coh.org (M.T.);; 2 Department of Hematology & Hematopoietic Cell Transplantation, City of Hope Comprehensive Cancer Center, Duarte, CA 91010, USA

**Keywords:** RNA, next-generation sequencing (NGS), fusion, hematological malignancies

## Abstract

Recurrent fusions drive the pathogenesis of many hematological malignancies. Compared to routine cytogenetic/fluorescence in situ hybridization (FISH) studies, the RNA-based next-generation sequencing (NGS) fusion assay enables the identification of both known and novel fusions. In many cases, these recurrent fusions are crucial for diagnosis and are associated with prognosis, relapse prediction, and therapeutic options. The aim of this study is to investigate the application of the RNA-based NGS fusion assay in hematological malignancies. Our study included 3101 cases with available fusion results, and a fusion event was identified in 17.6% of cases. The discordant rate between the RNA-based NGS fusion assay and cytogenetic/FISH studies was 36.3%. Further analysis of discordant cases indicated that, compared to cytogenetic/FISH studies, the RNA-based NGS fusion assay significantly improved the identification of cryptic fusion genes, such as *NUP98::NSD1*, *P2RY8::CRLF2*, and *KMT2A* fusions involving different partners. Additionally, our study identified 24 novel fusions and 16 cases with the simultaneous presence of two fusions. These additional findings from the RNA-based NGS fusion assay resulted in improved risk stratification, disease targeting and monitoring. In conclusion, our study demonstrates the feasibility and utility of an RNA-based NGS fusion assay for patients with hematological malignancies, suggesting that it may be essential for the routine clinical workup of these patients.

## 1. Introduction

Hematologic malignancies are heterogenous diseases that arise from the uncontrolled proliferation of clonal hematopoietic cells. A high frequency of gene rearrangements is identified in hematological malignancies, and a proportion of recurrent gene rearrangements play a pivotal role in the pathogenesis of a spectrum of myeloid and lymphoid neoplasms. In many cases, these recurrent gene rearrangements are crucial for diagnosis and are associated with prognosis, relapse prediction, and therapeutic targets [1,2,3,4]. Additionally, the definition and diagnosis of hematological malignancies are evolving. Both the current 5th edition of the World Health Organization Classification of Haematolymphoid Tumors and the International Consensus Classification (ICC) emphasize the integration of comprehensive genetic testing into the diagnosis, prognosis, and treatment of patients with hematological malignancies [3,4].

Currently, cytogenetic studies, fluorescence in situ hybridization (FISH), and reverse transcription polymerase chain reaction (RT-PCR) are the most widely used methods for identifying gene rearrangements in patients with hematologic malignancies. However, these methods have inherent limitations due to technical challenges. While cytogenetic studies can typically identify gene rearrangements, some are cryptic, or cytogenetically not visible [5,6]. FISH and RT-PCR, on the other hand, can only be tailored to diagnose individual specific gene rearrangements and are unable to identify novel fusions or all potential fusion partners. For instance, the *KMT2A* gene rearrangement is associated with 50 different fusion partners, and the presence of distinct *KMT2A* gene rearrangements has prognostic utility [7,8].

While DNA-based next-generation sequencing (NGS) assays have been widely applied in the clinical workup of patients with hematological malignancies, RNA-based NGS assays are typically considered complementary to existing DNA-based NGS assays [9,10]. RNA-based NGS fusion assays enable the detection of therapeutically and prognostically significant fusions, as well as the characterization of fusion partners and breakpoints in hematological malignancies. Additionally, RNA-based NGS fusion assays can identify novel fusions or fusions that may be missed by cytogenetic/FISH studies. [11,12]. Therefore, the purpose of this study is to investigate the application of RNA-based NGS fusion assays in patients with hematological malignancies.

## 2. Results

### 2.1. Case Cohort Characteristics

Our study included a total of 3101 cases with RNA-based NGS fusion studies. Among these, a positive fusion result was identified in 17.6% (545/3101) of cases. As shown in Table 1, positive fusion findings were most commonly identified in B-lymphoblastic leukemia (B-ALL) (31.0%; 191/616), followed by mixed phenotype acute leukemia (25.0%; 3/12), acute myeloid leukemia (AML) (23.2%; 319/1377), myeloid sarcoma (16.7%; 2/12), and T-lymphoblastic leukemia (T-ALL) (15.7%; 16/102). Positive fusion findings were less common among patients with myelodysplastic syndrome (MDS), myeloproliferative neoplasm (MPN), MDS/MPN, or myeloid neoplasm.

As demonstrated in Table 2, the mean age of patients with positive fusion findings was 46.6 years (range: 1–87 years), with 291 (53.4%) males and 254 (46.6%) females. The most common diagnoses were AML (319/545; 58.5%), followed by B-ALL (191/545; 35.1%) and T-ALL (16/545; 2.9%). Bone marrow aspirate was the most common specimen type, accounting for 68.3% (372/545), followed by peripheral blood specimens (30.4%; 166/545) and FFPE specimens (1.3%; 7/545). The average tumor content for positive fusion cases in our cohort is 47.4% (range: 0–98%). As mentioned above, although our validation assay indicates a minimum tumor content of 5%, positive fusion events were identified in 66 (66/545; 12.1%) cases with tumor content less than 5%. Corresponding cytogenetics and FISH studies were available in 84.8% (462/545) and 83.3% (454/545) positive cases, respectively.

### 2.2. Distribution of Fusions in Hematological Malignancies

**Figure 1:**
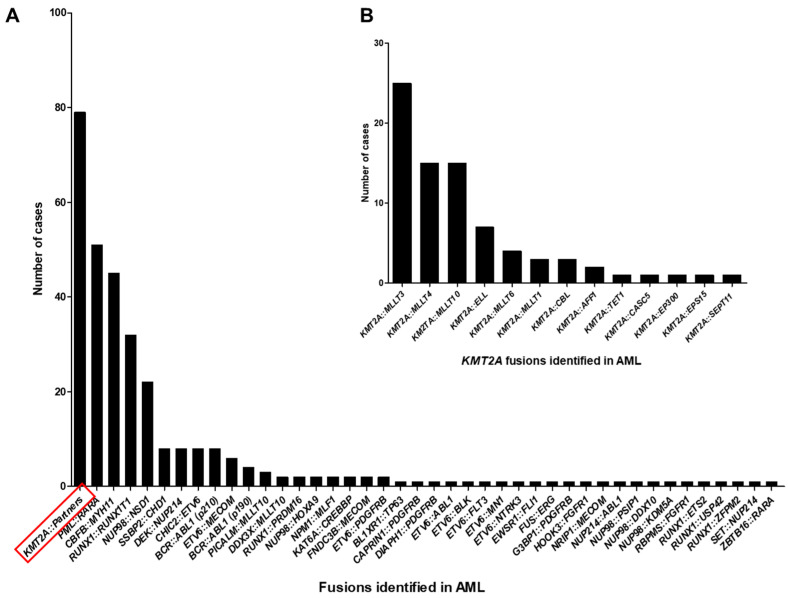
Frequency of fusions identified in acute myeloid leukemia (AML) (n = 319). (**A**). All fusions identified in AML; (**B**). *KMT2A* fusions with different partners identified in AML (n = 79) as shown in Figure (**A**), *KMT2A*::Partners.

As mentioned previously, positive fusion findings were identified in 319 AML cases. Interestingly, we found that *KMT2A* fusions (79/319; 24.8%) were the most common fusions identified in AML, followed by *PML::RARA* (51/319; 16.0%), *CBFB::MYH11* (45/319; 14.1%), *RUNX1::RUNX1T1* (32/319; 10.0%), and *NUP98::NSD1* (22/319; 6.9%) (Figure 1). According to the European Leukemia Net (ELN) 2023 risk classification for AML, 60.8% (194 out of 319) of these fusions were risk-relevant [13]. Among the 79 *KMT2A* fusions, 13 different partners were identified in our study, with the three most common being *KMT2A::MLLT3* (25/79; 31.6%), *KMT2A::MLLT4* (15/79; 19.0%) and *KMT2A::MLLT10* (15/79; 19.0%) (Figure 1B). Similar findings were reported by Meyer et al., with *KMT2A::MLLT3* and *KMT2A::MLLT10* being the most common fusions identified in AML [7].

**Figure 2:**
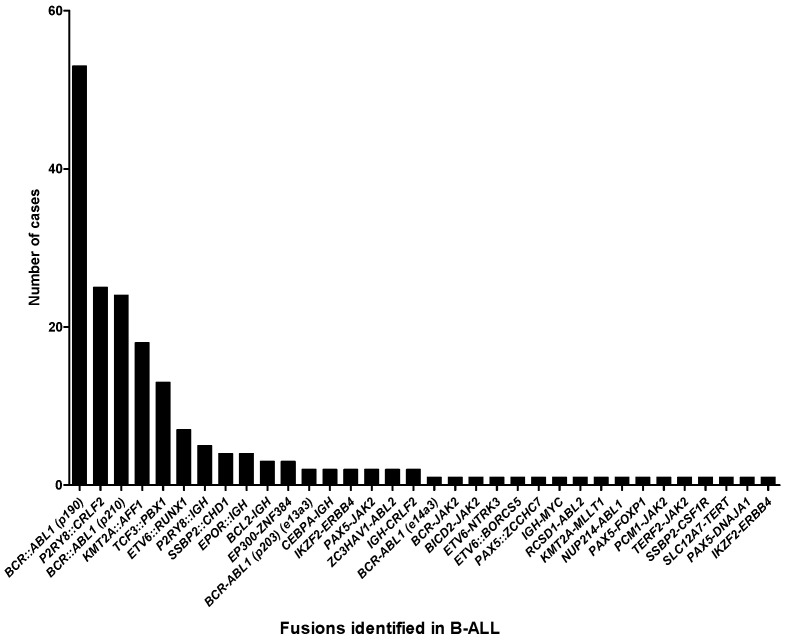
Frequency of fusions identified in B-lymphoblastic leukemia (n = 191).

**Figure 3:**
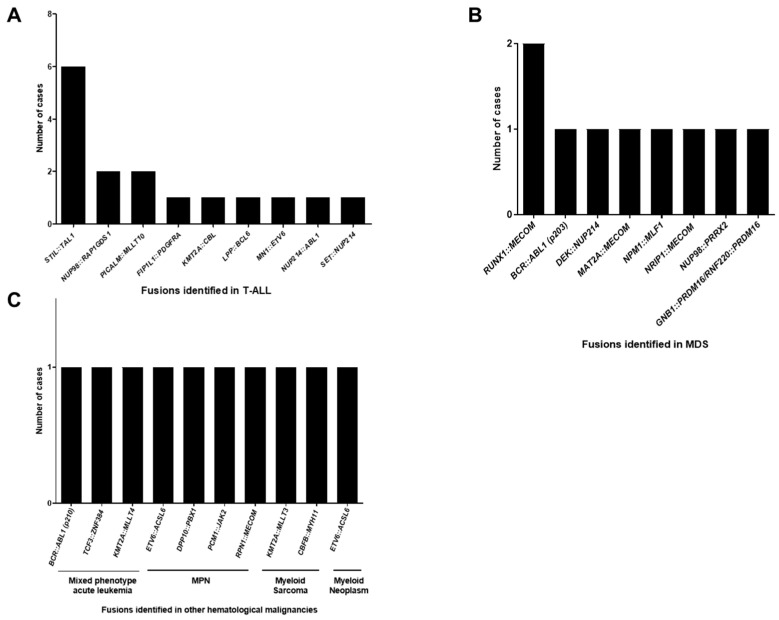
Frequency of fusions identified in other hematological malignancies. (**A**). T-lymphoblastic leukemia (n = 16); (**B**). Myelodysplastic syndrome (n = 9); (**C**). Mixed phenotype acute leukemia (n = 3), myeloproliferative neoplasm (n = 4), myeloid sarcoma (n = 1) and myeloid neoplasm (n = 2).

Furthermore, we investigated the prevalence of fusions among B-ALL patients. Figure 2 demonstrates that *BCR::ABL1* (p190) (53/191; 27.7%) was the most frequent fusions detected in B-ALL, followed by *P2RY8::CRLF2* (25/191; 13.1%), *BCR::ABL1* (p210) (24/191; 12.6%), *KMT2A::AFF1* (18/191; 9.4%) and *TCF3::PBX1* (13/191; 6.8%). Over 79% (151/191) of these fusions were associated with prognosis, as discussed by the 5th Edition of the World Health Organization Classification of Haematolymphoid Tumours: Lymphoid Neoplasms [2]. Additionally, for T-ALL patients, *STIL::TAL1* was the most common fusion detected in our cohort, with a ratio of 37.5% (6/16) (Figure 3A). The frequencies of fusions in MDS, mixed phenotype acute leukemia, MPN, myeloid sarcoma, and myeloid neoplasms are illustrated in Figure 3B,C.

### 2.3. Comparison of Fusions Detected by RNA-Based NGS Fusion Assay and Cytogenetic/FISH Studies

In the next step, we correlated the cytogenetic/FISH study findings with positive fusion cases. Among the 545 positive fusion cases, 89.2% (486/545) had available cytogenetic/FISH studies. In 63.7% (310/486) of cases, the findings were concordant between RNA-based NGS fusion assays and Cytogenetic/FISH studies. In 36.3% (171/468) of cases, RNA-based NGS fusions were positive, while the Cytogenetic/FISH studies were negative.

Next, we investigated the discordant cases between RNA-based NGS fusion assays and cytogenetic/FISH studies. As shown in Table 3, novel fusions were identified in 24 cases (24/545; 4.4%), with 21 of these cases having concurrent cytogenetic/FISH studies. Of these, only 5 (5/21; 23.8%) novel fusions were detected by cytogenetic/FISH studies. Additionally, the simultaneous presence of two fusions was observed in 16 cases (16/545; 2.9%), with 14 of these cases having concurrent cytogenetic/FISH studies. Only 5 (5/14; 35.7%) of these dual-fusion cases were identified by cytogenetic/FISH studies. The lists of these novel fusions and simultaneous dual fusions are provided in Table 3 and Table 4, respectively.

Table 5 summarizes the fusions, other than novel fusions or dual fusions, that were not detected by cytogenetic/FISH studies. We observed that 27 (27/171; 15.8%) cases had a tumor content less than 5%, which may partly explain why cytogenetic/FISH studies were negative in these cases. As listed in Table 5, no *NUP98* fusions (n = 23), including *NUP98::NSD1*, *NUP98::HOXA9*, *NUP98::DDX10* and *NUP98::KDM5A*, were identified by cytogenetic/FISH studies,. These findings are consistent with previous studies, which indicate that most *NUP98* fusions are cytogenetically normal [6,14,15].

As we all know, B-ALL patients with Ph-like features are associated with poor prognosis, and *P2RY8::CRLF2* and *EPOR::IGH* are the two most common fusions related to Ph-like B-ALL [16,17]. As indicated in Table 5, 85% of *P2RY8::CRLF2* (n = 17) and 100% *EPOR::IGH* (n = 4) fusions were not identified by cytogenetic/FISH studies. Additionally, *KMT2A* fusions have multiple known or novel partners, particularly *KMT2A::MLLT10* and *KMT2A::CBL*, which were often not identified by cytogenetic/FISH studies [5,18]. As shown in Table 5, 66.7% *KMT2A::CBL* (n = 2) and 41.7% *KMT2A::MLLT10* (n = 5) fusions were not detected by cytogenetic/FISH studies. We noted that one *KMT2A::CBL* case had a low tumor content of 1%, which could explain the discrepancy between RNA-based NGS fusion and cytogenetic/FISH results.

### 2.4. RNA-Based NGS Fusion Enables Monitoring in a Greater Proportion of Patients

Our laboratory has been able to monitor minimal residual disease (MRD) in hematological malignancies using a DNA-based NGS assay with a low allele frequency as low as 0.03%. As shown in Supplementary Figure S1, among the 545 cases with positive fusion findings, pathogenic/likely pathogenic mutations were identified in 85.2% (464/545) of cases, while 14.9% (81/545) had no pathogenic/likely pathogenic mutations identified, apart from the positive fusion findings. We observed that among the 464 cases with both positive fusion and pathogenic/likely pathogenic mutations, 1.7% (9/545) had pathogenic/likely pathogenic mutations limited to *DNMT3A*, *TET2*, or *ASXL1*. It is well known that *DNMT3A*, *TET2*, or *ASXL1* might not be good markers for MRD monitoring.

In addition, our cohort included four cases with *BCR::ABL1* (p203) fusion, which harbors a rare fusion transcript (e13a3). This transcript may not be detected by routine RT-PCR assay for p210/p190 due to primer-binding site mismatches. This rare p203 fusion, obtained through RNA-based NGS fusion assay with sequence information, is useful for monitoring the patients’ disease as well.

Therefore, by additionally using the RNA-based NGS fusion assay, we can identify sequence information from detected fusion genes to monitor the disease in a greater proportion of patients.

## 3. Discussion

Identification of fusions is crucial for the diagnosis, prognosis, and targeted therapy of patients with hematological malignancies. In our study, we investigated the feasibility and utility of an RNA-based NGS fusion assay for patients with hematological malignancies. We demonstrated that fusion events were identified in 17.6% of cases in our cohort, with even higher percentages observed in cases of AML, B-ALL, and mixed phenotype acute leukemia. Our results are promising and support the application of the RNA-based NGS fusion assays as part of the routine clinical workup for patients with suspected hematological malignancies.

Compared to other routine methods for detecting fusions, such as cytogenetics, FISH, or RT-PCR, the major advantage of the RNA-based NGS fusion assay is its capacity to identify cryptic fusions that are cytogenetically invisible, detect multiple fusions simultaneously, and fusions with novel partners, as observed in our cohort. In contrast, FISH is limited by the need for multiple sets of probes, which increases both the cost and turnaround time, potentially impacting the timeliness of clinical decision-making. Therefore, RNA-based NGS assays are more cost-effective for both patients and healthcare providers, due to their capacity for parallel sequencing of multiple genes. In our study, we found concordant results between RNA-based NGS fusion assay and cytogenetic/FISH studies in 63.7% cases. Discordant results, where the RNA-based NGS fusion was positive while cytogenetic/FISH studies were negative, were observed in 36.3% of cases. It appears that most of these discrepancies were related to the inability of cytogenetic/FISH studies to detect specific fusion transcripts.

For example, cytogenetic studies did not identify any *NUP98::NSD1* fusions in our study. Previous studies have shown that this fusion results from cryptic translocations not visible by conventional karyotyping [6,19,20]. The *NUP98::NSD1* fusion accounts for 16.1% of pediatric and 2.3% of adult cytogenetically normal AML patients. Additionally, this fusion has been identified as an independent factor to associated with poor clinical outcomes [6,21]. Preclinical studies have shown that leukemias carrying *NUP98*-gene rearrangements can potentially be targeted by menin inhibitors [22]. *P2RY8::CRLF2* fusions are frequently missed by conventional cytogenetic studies due to cryptic interstitial deletion. Ph-like ALL accounts for 15–30% of B-ALL cases, and *CRLF2* gene rearrangement is the most commonly defined subtype of Ph-like ALL, frequently partnering with either *P2RY8* or *IGH*. These cases are associated with a worse prognosis within Ph-like ALL [16,17,23,24,25]. Therefore, routine screening for these fusions at diagnosis is essential for proper identification and stratification of patients with B-ALL. The prognosis of acute leukemia with *KMT2A* gene rearrangement is poor, with a 5-year overall survival rate of less than 25% [26]. However, fusion transcripts involving *KMT2A* exhibit significant variability. In our cohort, we identified 79 patients with *KMT2A* fusions involving 13 different partners. *KMT2A::CBL*, *KMT2A::MLLT10* and *KMT2A::MLLT4* were the most common fusions missed by cytogenetic/FISH studies. Cryptic *KMT2A* fusions have been previously identified in several studies [27,28,29,30]. Additionally, *KMT2A::MLLT10*, known to comprise multiple exons of both *KMT2A* and *MLLT10* [7]. Currently, menin inhibitors are in clinical development for acute leukemia with *KMT2A* gene rearrangement [31]. A recent phase 1 clinical trial indicated that in children and adults with highly refractory acute leukemia with *KMT2A* gene rearrangement, menin inhibition with Revumenib monotherapy was associated with promising anti-leukemic activity, leading to deep and sustained remission [32]. Therefore, including RNA-based NGS fusion assay in the routine clinical workup for hematological malignancies is crucial.

In our study, novel fusions were identified in 4.4% (n = 24) of all positive cases. False positive events are common in RNA-based NGS fusion assays, primarily resulting from misalignment of read sequences due to polymorphisms, homology, and sequencing errors [33,34]. Therefore, all novel fusions need validation by orthogonal methods, and their presence in healthy populations should be investigated. Additionally, functional analysis is essential for accurately reporting these novel fusions, as it may help to better understand the pathogenesis of the disease.

Moreover, the RNA-based NGS assay allows for the identification of sequence information, particularly for rare fusions, such as *BCR::ABL1* p203, which cannot be detected by RT-PCR for *BCR::ABL1* p210/p190 due to primer-binding site mismatches. By providing the sequence information for these specific fusions, it was possible to design an RT-PCR based MRD assay. DNA or RNA based NGS assays are more recently developed methods for MRD detection. However, for fusion genes with highly expressed RNA levels, RNA-based NGS assays are preferable because the expression will result in more RNA copies of the fusion transcript compared to just one fusion DNA copy per cell [35,36,37].

Our study has some limitations. Firstly, our cohort includes three different versions of RNA-based NGS fusion assay. Although all three versions include the most important fusion genes for hematological malignancies, the data is not uniform across all cases in our cohort. Secondly, as our institution is a tertiary cancer center, there may be a selection bias toward more aggressive disease, and some cases may lack the initial molecular signature. Thirdly, we did not perform functional studies to further investigate the roles of the novel fusions.

In conclusion, our study demonstrates the utility and feasibility of incorporating the RNA-based NGS fusion panel for patients with suspected hematological malignancies in routine clinical settings. In particular, identifying cytogenetically cryptic fusions and fusions with multiple partners will improve risk stratification and ultimately benefit patients through tailored treatment options. Additionally, the discovery of rare or novel fusions will also aid in understanding the pathogenesis of the disease and ultimately improve patient outcomes.

## 4. Materials and Methods

### 4.1. Patients and Specimens

The study was approved by the City of Hope Comprehensive Cancer Center Review Board (IRB #15198). We conducted a retrospective review of patients with hematological malignancies who underwent one of the three versions of the RNA-based NGS fusion assay between August 2016 and February 2024 at the CLIA-approved clinical molecular diagnostics laboratory. Patients diagnosed with hematological malignancies at our institution, including acute leukemia, MDS, MPN, MDS/MPN, myeloid neoplasm and myeloid sarcoma, were consecutively included in our study. Patients’ demographic, pathological, and molecular features were collected through chart review.

### 4.2. RNA-Based NGS Fusion Assay

This RNA-based NGS assay detects potentially clinically actionable structural rearrangement events in cancers. It utilizes the Archer platform to detect gene rearrangements in 23 to 165 genes relevant to hematological malignancies, solid tumors, and sarcomas. The gene lists for the three different versions of RNA-based NGS fusion panels are summarized in Supplementary Table S1. Peripheral blood, bone marrow aspirates, and formalin-fixed paraffin-embedded (FFPE) specimens were used as inputs for the fusion studies, with a requirement of 200 ng RNA. The minimum tumor cell content is 5%, as established by our validation assay; however, positive fusion events were identified even in cases with less than 5% tumor cells.

The workflow includes RNA extraction and library preparation using the Archer FusionPlex reagent kit (Integrated DNA Technologies, Redwood City, CA, USA) for Illumina sequencing. The cDNA strands undergo end repair, adenylation, and ligation to half-functional universal adapters containing sample barcodes. Subsequently, two rounds of multiplex PCR are performed using gene-specific primers (GSP1 and GSP2) and a primer complementary to the universal adapter, enabling enrichment for target genes and identification of known and novel fusion partners. The target amplicon library is then quantitated and sequenced using the Illumina MiSeq system (Illumina, San Diego, CA, USA) and the data are analyzed using Archer FusionPlex Software version 7.3.2 (Integrated DNA Technologies, Redwood City, CA, USA).

Each run of the RNA-based NGS fusion assay includes both a positive control and a negative control. The positive control, termed “Multiplexed Reference Controls,” consists of SeraCare reference material containing 18 confirmed fusions and oncogenic isoforms with varying fusion transcript levels. This positive control is used for ongoing monitoring of the RNA-based NGS assay to ensure accuracy and sensitivity. The negative control, referred to as the No Template Control, comprises a tube containing all reaction components except RNA. This control is employed to assess potential cross-contamination during the assay. Additionally, each step of the assay includes a specific quality control (QC) evaluation, covering the nucleic acid extraction, library preparation, sequencing, and Archer analysis steps. These QC checks verify that all metrics meet the acceptable criteria established in our laboratory’s standard operating procedures. Finally, the sequencing data is reviewed by curators and pathologists prior to the release of final reports.

The RNA-based NGS fusion panel Version 1 was applied to 286 cases (August 2016 to December 2017), Version 2 to 1707 cases (January 2017 to February 2022), and Version 3 to 1108 cases (March 2022 to February 2024). The overall RNA-based NGS fusion success rate is more than 99.5%.

### 4.3. Statistical Analysis

Baseline characteristics are presented as mean and range for continuous variables, and frequency for categorical variables.

## Figures and Tables

**Table 1:** Characteristics of patients with hematological malignancies in our cohort (n = 3101).

Disease Type (n, %)			
	Total	Positive	Negative
	(n = 3101)	(n = 545)	(n = 2556)
AML	1377	319 (23.2%)	1058 (76.8%)
B-ALL	616	191 (31.0%)	425 (69.0%)
T-ALL	102	16 (15.7%)	86 (84.3%)
Mixed phenotype acute leukemia	12	3 (25.0%)	9 (75.0%)
MDS	455	9 (2.0%)	446 (98.0%)
MPN	440	4 (0.9%)	436 (99.1%)
MDS/MPN	58	0 (0%)	58 (100%)
Myeloid neoplasm	29	1 (3.4%)	28 (96.6%)
Myeloid sarcoma	12	2 (16.7%)	10 (83.3%)

Abbreviations: AML, Acute myeloid leukemia; B-ALL, B-lymphoblastic leukemia; T-ALL, T-Lymphoblastic leukemia; MDS, Myelodysplastic syndrome; MPN, Myeloproliferative neoplasm.

**Table 2:** Characteristics of patients with positive fusion findings (n = 545).

Characteristics	Value
Age, mean (range), y	46.6 (1–87)
Sex, n (%)	
Male	291 (53.4%)
Female	254(46.6%)
Diagnosis, n (%)	
AML	319 (58.5%)
B-ALL	191 (35.1%)
T-ALL	16 (2.9%)
Mixed phenotype acute leukemia	3 (0.6%)
MDS	9 (1.6%)
MPN	4 (0.7%)
Myeloid neoplasm	2 (0.4%)
Myeloid sarcoma	1 (0.2%)
Specimen Type, n (%)	
Bone Marrow Aspirate	372 (68.3%)
Peripheral Blood	166 (30.4%)
Bone marrow clot sections (FFPE)	7 (1.3%)
Tumor content (%), mean (range)	0–98% (47.4%)
Low tumor content (<5%), n (%)	66 (12.1%)
Analyzed with Cytogenetics/FISH Studies	
Cytogenetics, n (%)	462 (84.8%)
FISH, n (%)	454 (83.3%)

Abbreviations: AML, Acute myeloid leukemia; B-ALL, B lymphoblastic leukemia; FFPE; Formalin fixed paraffin embedded; FISH, Fluorescence in situ hybridization; MDS, Myelodysplastic syndrome; MPN, Myeloproliferative neoplasm; T-ALL, T lymphoblastic leukemia.

**Table 3:** Lists of novel fusions identified in our cohort (n = 24).

Novel Fusions	Diagnosis	Case Number	Identified by Cytogenetic/FISH Studies
*ATP8B4::MECOM*	AML	1	Yes
*BANP::RARA*	AML	1	Yes
*ELF1::PRDM16*	AML	1	Yes
*LARP1::NUTM1*	AML	1	Yes
*XBP1::JAK2*	B-ALL	1	Yes
*ARHGAP15::NUTM1*	AML	1	No
*ATXN3::ETV6*	AML	1	No
*BCL11B::DEK*	AML	1	No
*ELF1::MECOM*	AML	1	No
*ENO1::PRDM16*	AML	1	No
*EPOR::ANKRD24*	AML	1	No
*ETV6::BLK*	AML	1	No
*GABPB1::NUTM1*	AML	1	No
*GNB1::PRDM16*	MDS	1	No
*IQGAP2::FLT3*	AML	1	No
*MAP4K4::ABL1*	B-ALL	1	No
*PAX5::GNE*	B-ALL	1	No
*RCAN1::BCL2*	B-ALL	1	No
*RNF220::PRDM16*	AML	1	No
*SETD2::MME*	B-ALL	1	No
*TCF12::DMXL2*	AML	1	No
*ACTB::MYC*	B-ALL	1	N/A
*DPP10::PBX1*	MPN	1	N/A
*SNAPC4::NOTCH1*	B-ALL	1	N/A

Abbreviations: AML, Acute myeloid leukemia; B-ALL, B-acute lymphoblastic leukemia; MDS, Myelodysplastic syndrome; MPN, myeloproliferative neoplasm; N/A, Not applicable.

**Table 4:** Lists of dual fusions identified in our cohort (n = 16).

Novel Fusions	Diagnosis	Case Number	Identified by Cytogenetic/FISH Studies
*BCR::ABL1* (p190) & *ETV6::NTRK3*	AML	1	Yes
*CBFB::MYH11* & *SSBP2::CHD1*	AML	1	Yes
*KMT2A::MLLT10* & *SSBP2::CHD1*	AML	1	Yes
*KMT2A::MLLT10* & *TBL1XR1::TP63*	AML	1	Yes
*KMT2A::MLLT4* & *ZBTB16::RARA*	AML	1	Yes
*BCR::ABL1* (p190) & *RCAN1::BCL2*	B-ALL	1	No
*BCR::ABL1* (p190) & *SLC12A7::TERT*	B-ALL	1	No
*BCR::ABL1* (p210) & *NUP98::PSIP1*	AML	1	No
*ETV6::ABL1* & *NUP98::NSD1*	AML	1	No
*ETV6::BORCS5* & *PAX5::ZCCHC7*	B-ALL	1	No
*GNB1::PRDM16* & *RNF220::PRDM16*	MDS	1	No
*HOOK3::FGFR1* & *CHIC2::ETV6*	AML	1	No
*IGH::CRLF2* & *P2RY8::IGH*	B-ALL	1	No
*RUNX1:: ETS2* & *ETV6::MECOM*	AML	1	No
*BCR::ABL1* (p190) & *NUP98::NSD1*	AML	1	N/A
*ETV6::MN1* & *CHIC2::ETV6*	AML	1	N/A

Abbreviations: AML, Acute myeloid leukemia; B-ALL, B-acute lymphoblastic leukemia; MDS, Myelodysplastic syndrome; N/A, Not applicable.

**Table 5:** Discordant cases between RNA-based NGS fusion assay and Cytogenetic/FISH studies (n = 171).

Discordant Cases	Detected by RNA-Based Fusion Assay Only	Detected by RNA-Based Fusion Assay and Cytogenetics/FISH
Novel fusion with available cytogenetic/FISH studies (n = 21), n (%)	16 (76.2%)	5 (23.8%)
Dual fusions with available cytogenetic/FISH studies (n = 14), n (%)	9 (66.7%) *	5 (33.3%)
Low tumor content (<5%)	27	30
Tumor content (mean, range)	1.1% (0–4.5%)	1.79% (0–4.5%)
List of fusions, n (%)	121 (70.8%)	N/A
*NUP98::NSD1*; *NUP98::HOXA9*; *NUP98::DDX10*; *NUP98::KDM5A*	19 (100%); 2 (100%); 1 (100%); 1 (100%)	0 (0%)
*P2RY8::CRLF2*	17 (85%)	3 (15%)
*KMT2A::MLLT10*; *KMT2A::CBL*; *KMT2A-MLLT4*; *KMT2A::ELL*	5 (41.7%); 2 (66.7%); 2 (20%); 1 (16.7%)	7 (58.3%); 1 (33.3%); 8 (80%); 5 (83.3%)
*SSBP2::CHD1*	8 (100%)	0 (0%)
*CHIC2::ETV6*	5 (100%)	0 (0%)
*STIL::TAL1*	5 (100%)	0 (0%)
*EPOR::IGH*	4 (100%)	0 (0%)
*EP300::ZNF384*	3 (100%)	0 (0%)
*IKZF2::ERBB4*	3 (100%)	0 (0%)
*P2RY8::IGH*	3 (75%)	1 (25%)
*DDX3X::MLLT10*	2 (100%)	0 (0%)
*ETV6::MECOM*; *FNDC3B::MECOM*; *NRIP1::MECOM*	2 (33.3%); 2 (100%); 1 (100%)	4 (66.7%); 0 (0%); 0 (0%)
*PAX5::JAK2*; *PAX5::DNAJA1*; *PAX5::FOXP1*	2 (100%); 1 (100%); 1 (100%)	0 (100%); 0 (0%); 0 (0%)
*RUNX1::RUNX1T1*; *RUNX1::PRDM16*; *RUNX1::USP42*	3 (10%); 1 (50%); 1 (100%)	27 (90%); 1 (50%); 0 (0%)
*SET::NUP214*	2 (100%)	0 (0%)
*TCF3::PBX1*; *TCF3::ZNF384*	2 (20%); 1 (100%)	8 (80%); 0 (0%)
*PCM1::JAK2*; *BICD2::JAK2*; *TERF2::JAK2*	1 (100%); 1 (100%); 1 (100%)	0 (0%); 0 (0%); 0 (0%)
*CAPRIN1::PDGFRB*; *DIAPH1::PDGFRB*	1 (100%); 1 (100%)	0 (0%); 0 (0%)
*CBFB::MYH11*	1 (2.5%)	39 (97.5%)
*BCR::ABL1* (p203)	1 (100%)	0 (0%)
*ETV6::NTRK3*	1 (100%)	0 (0%)
*FUS::ERG*	1 (100%)	0 (0%)
*GABPB1::NUTM1*	1 (100%)	0 (0%)
*KAT6A::CREBBP*	1 (100%)	0 (0%)
*LPP::BCL6*	1 (100%)	0 (0%)
*NUP214::ABL1*	2 (100%)	0 (0%)
*PICALM::MLLT10*	2 (100%)	0 (0%)
*RBPMS::FGFR1*	1 (100%)	0 (0%)
*RCSD1::ABL2*	1 (100%)	0 (0%)
*SSBP2::CSF1R*	1 (100%)	0 (0%)

*, Two dual fusion cases include novel fusions as well. Abbreviations: FISH, fluorescence in situ hybridization; N/A, not applicable.

## Data Availability

The data presented in this study are available on request from the corresponding author.

## Supplementary Materials

The following supporting information can be downloaded at: https://www.mdpi.com/article/10.3390/ijms26020435/s1.
