# Supplementary material for: Application of RNA-Based Next-Generation Sequencing Fusion Assay for Hematological Malignancies
Source: Int J Mol Sci. 2025 Jan 7;26(2):435. doi: 10.3390/ijms26020435 (PMC11765404; doi:10.3390/ijms26020435)

Supplementary Table S1A. RNA-based NGS fusion panel gene list (Version 1).

**RNA-based fusion panel: 23 genes**						
ABL1	*ABL2*	*ALK*	*BCR*	*CBFB*	*CRLF2*	*CSF1R*
FGFR1	*JAK2*	*KMT2A*	*MECOM*	*MKL1*	*NOTCH1*	*NUP214*
PDGFRA	*PDGFRB*	*PICALM*	*RARA*	*RBM15*	*RUNX1*	*RUNX1T1*
TAL1	*TCF3*					

Supplementary Table S1B. RNA-based NGS fusion panel gene list (Version 2).

**RNA-based fusion panel: 74 genes**						
ABL1	*CCND3*	*EBF1*	*JAK2*	*MYC*	*PAX5*	*RARA*
ABL2	*CDK6*	*EIF4A1*	*KAT6A*	*MYH11*	*PBX1*	*RBM15*
ALK	*CHD1*	*EPOR*	*KLF2*	*NF1*	*PDCD1LG2*	*ROS1*
BCL11B	*CHIC2*	*ERG*	*KMT2A*	*NFKB2*	*PDGFRA*	*RUNX1*
BCL2	*CIITA*	*ETV6*	*MALT1*	*NOTCH1*	*PDGFRB*	*RUNX1T1*
BCL6	*CREBBP*	*FGFR1*	*MECOM*	*NTRK3*	*PICALM*	*SEMA6A*
BCR	*CRLF2*	*GLIS2*	*MKL1*	*NUP214*	*PML*	*SETD2*
BIRC3	*CSF1R*	*IKZF1*	*MLF1*	*NUP98*	*PRDM16*	*STIL*
CBFB	*DEK*	*IKZF2*	*MLLT10*	*P2RY8*	*PTK2B*	*TAL1*
CCND1	*DUSP22*	*IKZF3*	*MLLT4*	*PAG1*	*P2PR8*	*TCF3*
TFG	*TP63*	*TYK2*	*ZCCHC7*			

Supplementary Table S1C. RNA-based NGS fusion panel gene list (Version 3).

**RNA-based fusion panel: 165 genes**														
ABL1	*ABL2*	*AKT3*	*ALK*	*ARHGAP26*	*AXL*	*BCL11B*	*BCL2*	*BCL6*	*BCOR*	*BCR*	*BIRC3*	*BRAF*	*BRD3*	*BRD4*
CAMTA1	*CBFB*	*CCNB3*	*CCND1*	*CCND3*	*CD151*	*CDK6*	*CHD1*	*CHIC2*	*CIC*	*CIITA*	*CREBBP*	*CRLF2*	*CSF1R*	*DEK*
DUSP22	*EBF1*	*EGFR*	*EIF4A1*	*EPC1*	*EPOR*	*ERG*	*ESR1*	*ESRRA*	*ETV1*	*ETV4*	*ETV5*	*ETV6*	*EWSR1*	*FGFR1*
FGFR2	*FGFR3*	*FGR*	*FOSB*	*FOXO1*	*FUS*	*GLI1*	*GLIS1*	*HMGA2*	*IKZF1*	*IKZF2*	*IKZF3*	*IL2RB*	*INSR*	*JAK2*
JAZF1	*KAT6A*	*KLF2*	*KMT2A*	*LMO2*	*LYN*	*MALT1*	*MAML2*	*MAN2B1*	*MAST1*	*MAST2*	*MBTD1*	*MEAF6*	*MECOM*	*MFE2D*
MET	*MGEA5*	*MKL1*	*MKL2*	*MLF1*	*MLLT10*	*MLTT4*	*MN1*	*MSMB*	*MUSK*	*MYB*	*MYC*	*MYH11*	*NCOA1*	*NCOA2*
NF1	*NFKB2*	*NOTCH1*	*NOTCH2*	*NPM1*	*NR4A3*	*NRG1*	*NTRK1*	*NTRK2*	*NTRK3*	*NUMBL*	*NUP214*	*NUP98*	*NUTM1*	*P2RY8*
PAG1	*PAX3*	*PAX5*	*PAX7*	*PBX1*	*PDCDILG2*	*PDGFB*	*PDGFRA*	*PDGFRB*	*PHF1*	*PICALM*	*PIK3CA*	*PKN1*	*PLAG1*	*PML*
PPARG	*PRDM16*	*PRKCA*	*PRKCB*	*PRKD1*	*PRKD2*	*PRKD3*	*PTK2B*	*RAF1*	*RANBP17*	*RARA*	*RBM15*	*RECK*	*RELA*	*RET*
ROS1	*RSPO2*	*RSPO3*	*RUNX1*	*RUNX1T1*	*SEMA6A*	*SETD2*	*SS18*	*STAG2*	*STAT6*	*STIL*	*TAF15*	*TAL1*	*TCF12*	*TCF3*
TERT	*TFE3*	*TFEB*	*TFG*	*THADA*	*TMPRSS2*	*TP63*	*TSLP*	*TYK2*	*USP6*	*YAP1*	*YWHAE*	*ZCCHC7*	*ZMYND11*	*ZNF384*

Supplementary Figure S1. Impacts of RNA-based next-generation fusion assay for disease monitoring.

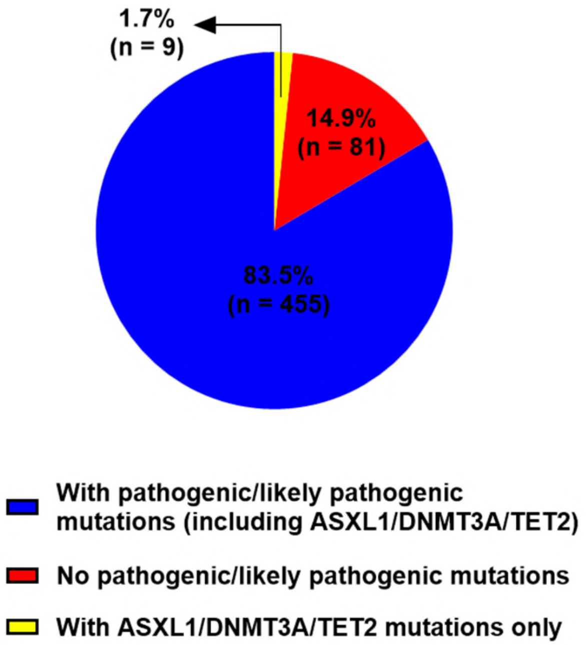

Supplement: Supplementary file 1 [file ijms-26-00435-s001.zip › ijms-3372926-supplementary.pdf]
